# Micro-pharmacokinetics: Quantifying local drug concentration at live cell membranes

**DOI:** 10.1038/s41598-018-21100-x

**Published:** 2018-02-22

**Authors:** Karolina Gherbi, Stephen J. Briddon, Steven J. Charlton

**Affiliations:** 1 Division of Pharmacology, Physiology and Neuroscience, School of Life Sciences, Medical School, University of Nottingham, Queen’s Medical Centre, Nottingham, NG7 2UH UK; 2 Centre of Membrane Proteins and Receptors, Universities of Birmingham and Nottingham, The Midlands, UK; 3 Excellerate Bioscience Ltd, MediCity, Nottingham, NG90 6BH UK

## Abstract

Fundamental equations for determining pharmacological parameters, such as the binding affinity of a ligand for its target receptor, assume a homogeneous distribution of ligand, with concentrations in the immediate vicinity of the receptor being the same as those in the bulk aqueous phase. It is, however, known that drugs are able to interact directly with the plasma membrane, potentially increasing local ligand concentrations around the receptor. We have previously reported an influence of ligand-phospholipid interactions on ligand binding kinetics at the β_2_-adrenoceptor, which resulted in distinct “micro-pharmacokinetic” ligand profiles. Here, we directly quantified the local concentration of BODIPY630/650-PEG8-*S*-propranolol (BY-propranolol), a fluorescent derivative of the classical β-blocker propranolol, at various distances above membranes of single living cells using fluorescence correlation spectroscopy. We show for the first time a significantly increased ligand concentration immediately adjacent to the cell membrane compared to the bulk aqueous phase. We further show a clear role of both the cell membrane and the β_2_-adrenoceptor in determining high local BY-propranolol concentrations at the cell surface. These data suggest that the true binding affinity of BY-propranolol for the β_2_-adrenoceptor is likely far lower than previously reported and highlights the critical importance of understanding the “micro-pharmacokinetic” profiles of ligands for membrane-associated proteins.

## Introduction

The fundamental equations routinely used in the determination of pharmacological parameters, such as binding affinities, assume a freely diffusible and homogeneously distributed ligand in solution. In experimental terms this means that the ligand concentration near the receptor is assumed to be the same as in the bulk aqueous phase. It is, however, known that drugs are able to interact directly with the plasma membrane^1–5^, and ligands that are able to partition into the lipid bilayer have been proposed to cause higher local ligand concentrations in the membrane^1,2^. Ligand diffusion in and out of these ligand depots in the membrane, and ligand interactions at the membrane/water interface have also been proposed to potentially increase ligand concentrations in the immediate vicinity of the membrane compared to the bulk aqueous phase^6^, although ligand concentrations in such defined localisations have never been experimentally measured before. We have recently reported a direct correlation of greater levels of ligand-phospholipid interactions and faster association rates for a range of β_2_-adrenoceptor ligands^5^, further suggesting the presence of higher local concentrations near the membrane due to the concentration-dependent nature of ligand association rates. The β-adrenoceptor antagonist propranolol has been shown to make hydrophobic and electrostatic interactions with phospholipid tails and head groups in the membrane^7,8^, although it is described to access the β_2_-adrenoceptor binding pocket from the aqueous solution above the extracellular surface of the receptor^9^.

In this study, we aimed to address this hypothesis of higher local drug concentrations, quantifying concentrations of the fluorescent propranolol derivative BODIPY630/650-PEG8-*S*-propranolol (BY-propranolol)^10^ immediately above the cell membrane and the bulk aqueous phase using fluorescence correlation spectroscopy (FCS). FCS measures fluorescence intensity fluctuations of a fluorescent species diffusing through a small (~0.25 fL) confocal volume over time, with autocorrelation analysis of these fluctuations allowing quantification of the concentration and diffusion coefficient of the fluorescent species under investigation^11^. This single molecule detection technique has been used in highly localised subcellular compartments to gain spatiotemporal resolution of the dynamic interactions of fluorescently labelled molecules, such as lipids^12^, transcription factors^13^, cytoplasmic proteins^14^, membrane associated proteins^15,16^ and small molecule ligands^17,18^. Here we use FCS to show that BY-propranolol concentrations in the immediate vicinity of the membranes of individual CHO cells are substantially higher than those in the bulk solution phase, and that these concentrations are influenced by the presence of the β_2_-adrenoceptor.

## Results

### Measuring local BY-propranolol concentrations above membranes of single cells

**Figure 1:**
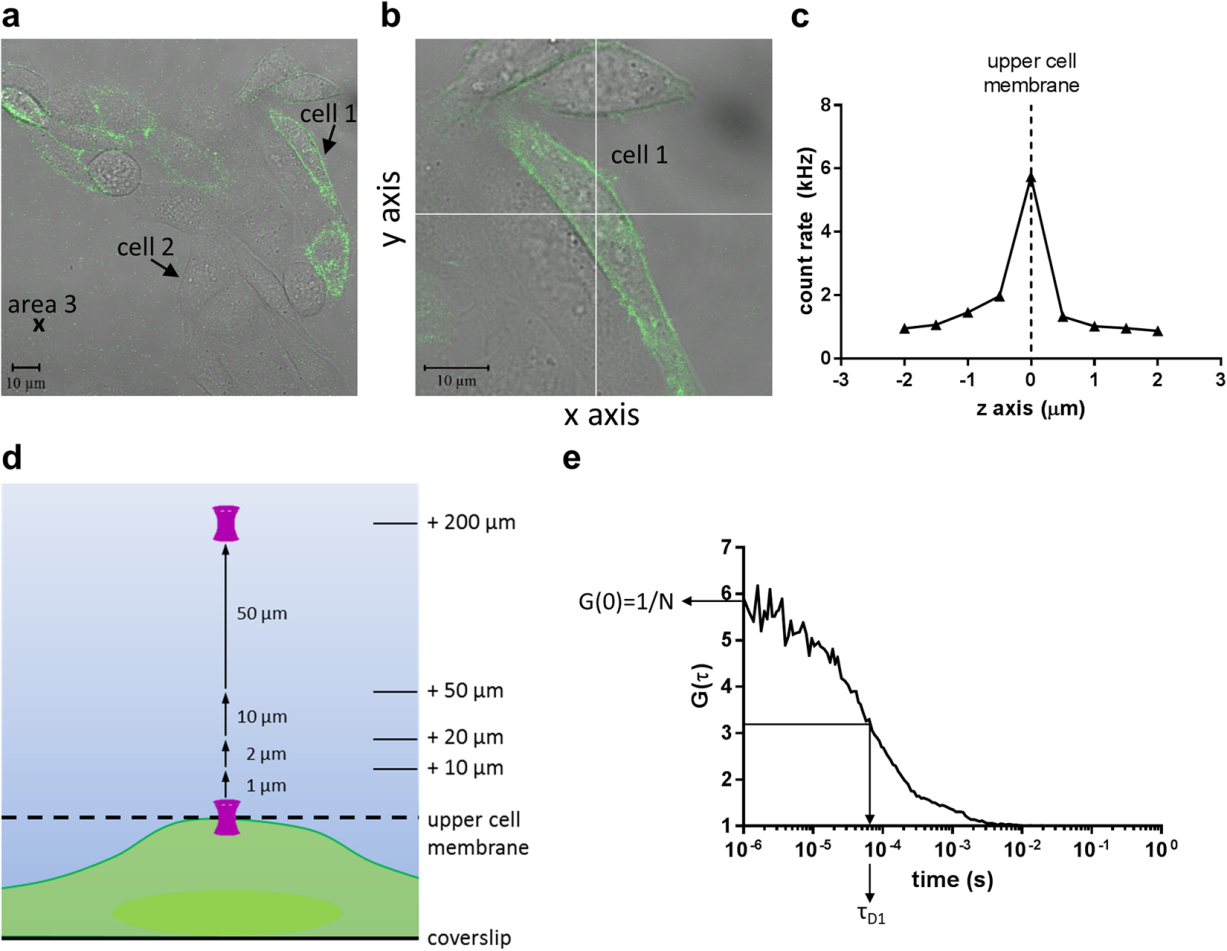
FCS experimental set-up used in this study. (**a**) Confocal image of receptor expressing (cell 1) and non-receptor expressing (cell 2) CHO-β_2_GFP cells, and control areas of no cell (area 3) used for FCS measurements. (**b**) Crosshair placement over the nucleus of a single cell was used to define the positioning of the FCS confocal volume in the x-y plane. (**c**) Detection of BY-propranolol fluorescence intensities in the z dimension allowed localisation of the upper cell membrane. (**d**) Schematic representation of confocal volume positions covering a range of distances from 2–200 µm above the upper membrane of a single cell in 1, 2, 10 and 50 μm steps. (**e**) Determination of particle number (N) and dwell time (τ_D1_) to quantify concentration and diffusion coefficient of BY-propranolol from its FCS autocorrelation curve.

**Figure 2:**
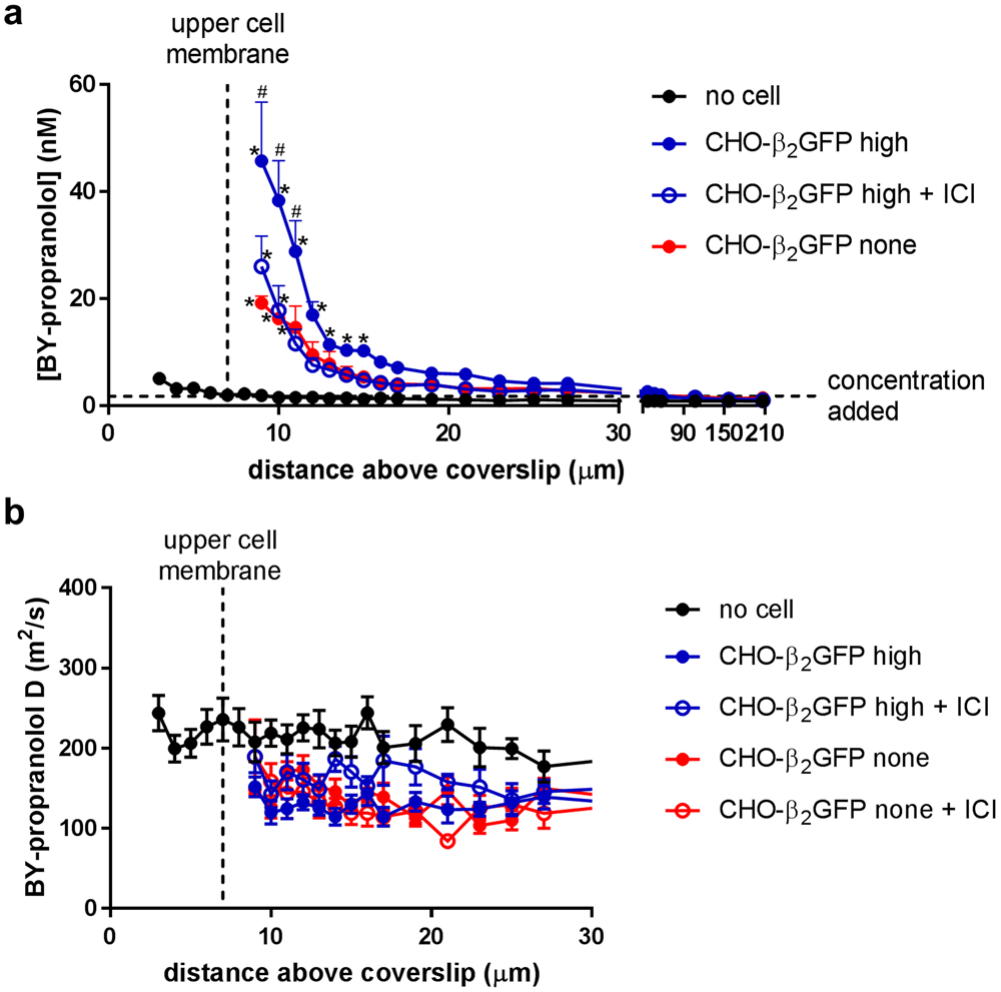
Role of the presence of the target receptor and the cell membrane on local BY-propranolol concentrations and diffusion coefficients. (**a**,**b**) Local concentrations (**a**) and diffusion coefficients (**b**) of BY-propranolol measured 2–200 µm above membranes of receptor expressing (high) CHO-β_2_GFP cells in the absence (n = 13) and presence of 550 nM ICI 118,551 (ICI; n = 7) and non-receptor expressing (none) CHO-β_2_GFP cells (n = 10) following 2 hours BY-propranolol incubation. The same experiments were carried out in areas of no cell (3–207 µm above the coverslip; absence of antagonist only; n = 8). Data shown are mean ± s.e.m. of *n* individual cells investigated on the same number of separate experimental days, and ^#^denotes statistical significance (*P* < 0.05) of the value determined in receptor-expressing cells (CHO-β_2_GFP high) compared to the value determined in no cells, non-receptor expressing (CHO-β_2_GFP none) cells and receptor-expressing cells in the presence of ICI 118,551 at an equivalent distance from the coverslip (2-way ANOVA, Tukey’s post hoc test), whilst * Indicates statistically significant differences (*P* < 0.05) in ligand concentrations at various distances above the coverslip compared to the concentration determined at the furthest distance measured for each individual condition (2-way ANOVA, Tukey’s post hoc test). BY-propranolol diffusion coefficients were not statistically different across the range of distances tested (P > 0.05, 2-way ANOVA, Tukey’s post hoc test).

In order to determine ligand concentrations in the vicinity of individual cells, we used the technique of FCS. FCS uses a small defined detection volume of ~0.25 fL (0.2 × 1 μm) allowing a high spatial resolution to measurements of concentration. To investigate the distribution of drug concentration in the measurement well, we first measured BY-propranolol concentrations in solution in the absence of any cells at a range of distances from 3 µm to 207 µm above the glass coverslip (Fig. 1) to establish whether the measured concentration reflected the added concentration of 1.8 nM BY-propranolol (circa 1× K_D_; Extended Data Fig. 1) in these distinct locations. Autocorrelation analysis of BY-propranolol fluorescence fluctuations detected in the FCS detection volume placed 3, 4 and 5 µm above the coverslip revealed concentrations of 5.1 ± 0.8 nM (n = 8), 3.2 ± 0.4 nM (n = 8) and 3.3 ± 0.5 nM (n = 8), respectively, all of which were higher than the 1.8 nM BY-propranolol concentration added (*P* < 0.05, t-test). In contrast, concentrations at distances greater than 5 µm above the coverslip were not significantly different from the added concentration (*P* > 0.05, t-test), but showed a steady decline with increasing distance to 0.8 ± 0.1 nM (n = 8) measured 207 µm above the coverslip (Fig. 2a).

We next aimed to determine the BY-propranolol concentrations above membranes of cells from a stable mixed population of CHO-β_2_GFP cells that allowed the selection of non-receptor and receptor expressing cells based on the detection of GFP fluorescence (Extended Data Fig. 2). FCS measurements taken at 2 µm above the membrane of non-receptor expressing cells determined a BY-propranolol concentration of 19.2 ± 1.3 nM (n = 10), which was markedly higher than the concentration measured nearest the coverslip in the absence of cells (*P* < 0.05, two-way ANOVA). Furthermore, a steeper concentration gradient was observed, with ligand concentrations declining to 1.5 ± 0.3 nM (n = 10) in the bulk aqueous phase 200 µm above cell membranes (Fig. 2a).

We then hypothesised that ligand concentrations near the membrane might be increased further in the presence of target receptors that facilitate specific ligand binding interactions^6^. To test this, we assessed BY-propranolol concentrations above membranes of cells with detectable β_2_GFP expression, as determined by GFP fluorescence intensity measurements (see Extended Data Fig. 2). Interestingly, the BY-propranolol concentration measured 2 µm above the membrane of these cells was higher (45.7 ± 11.0 nM, n = 13) than the concentration obtained in cells without detectable receptor expression (*P* < 0.05, two-way ANOVA). To confirm that these findings were receptor-driven in a specific manner, we examined the effects of the β_2_-adrenoceptor selective antagonist ICI 118,551 (550 nM, 10 min pre-incubation, 22 °C) on local BY-propranolol concentrations in the same cells. Indeed, ligand concentrations 2 µm above cell membranes were reduced in the presence of ICI 118,551 (27.1 ± 6.4 nM, n = 7). In cells with no detectable β_2_GFP expression BY-propranolol concentrations were unchanged in the presence of ICI 118,551 (Extended Data Fig. 3a). These data support the notion that the local BY-propranolol concentration in the immediate vicinity of the cell membrane is influenced by specific β_2_-adrenoceptor interactions. Whilst no direct ligand-receptor interactions were measured at a distance of 2 µm above the cell membrane, our data nevertheless highlight a receptor-driven component in the determination of local BY-propranolol concentrations that is blocked in the presence of an antagonist. Furthermore, BY-propranolol diffusion co-efficients were similar over all distances in all conditions tested in this study (Fig. 2b, Extended Data Fig. 3b), ruling out potential effects due to different diffusion characteristics near the membrane environment compared to the bulk aqueous phase.

### Investigating local BY-propranolol concentration over time

**Figure 3:**
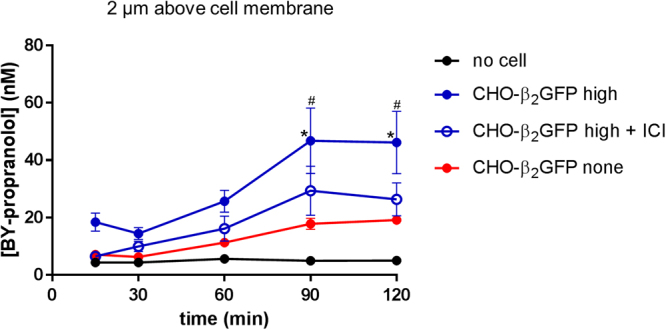
Effect of incubation time on BY-propranolol concentrations in the immediate vicinity of cell membranes. Local concentrations of BY-propranolol 2 µm above membranes of receptor expressing (high) CHO-β_2_GFP cells in the absence (n = 13) and presence of 550 nM ICI 118,551 (ICI; n = 7) and non-receptor expressing (none) CHO-β_2_GFP cells (n = 10) following a 15, 30, 60, 90 and 120 minutes BY-propranolol incubation. The same experiments were carried out in areas of no cell (data from measurements 3 µm above the coverslip; absence of antagonist only; n = 8). Data shown are mean ± s.e.m. of *n* individual cells investigated on the same number of separate experimental days, and ^#^denotes statistical significance (*P* < 0.05) of the value determined in receptor-expressing cells (CHO-β_2_GFP high) compared to the value determined in no cells at an equivalent time point (2-way ANOVA, Tukey’s post hoc test). *Indicates statistically significant differences (*P* < 0.05) in ligand concentrations compared to the concentration determined at the 15 minute time point (2-way ANOVA, Tukey’s post hoc test).

To gain further insight into what might influence local drug concentrations, we examined BY-propranolol concentration at a fixed distance of 2 µm above cell membranes over a time scale of 15 minutes to 2 hours, as all previously described experiments were performed following a 2 hour incubation of BY-propranolol. In the absence of cells, BY-propranolol concentrations measured closest to the coverslip did not change throughout the time course (Fig. 3), eliminating the possibility of non-cell related artefacts over time. Measurements above membranes of cells of no detectable receptor expression revealed BY-propranolol concentration increases in a linear fashion over time, although the concentration differences observed at the different time points were not statistically significant (*P* > 0.05, two-way ANOVA). In contrast, above membranes of receptor expressing cells BY-propranolol concentrations were significantly increased at 90 and 120 minutes compared to the first measurements taken at 15 and 30 minutes (*P* < 0.05, two-way ANOVA; Fig. 3a). This delay in increased local BY-propranolol concentrations may, at least in part, be related to its dissociation rate from the receptor (k_off_ 0.04 min^−1^, t_1/2_ 17 min; Extended Data Fig. 1b) and rebinding events whereby dissociated ligands bind neighbouring receptors instead of diffusing away into the bulk aqueous phase^6,19^, thus contributing to localising ligand concentrations near the membrane in a second order kinetic process^19^. In the same cells, in the presence of ICI 118, 551, BY-propranolol concentration increased in a similar pattern as detected in non-receptor expressing cells (Fig. 3a), confirming a receptor-driven influence on local ligand concentrations. The presence of ICI 118,551 did not affect BY-propranolol concentrations in cells with no detectable β_2_GFP expression (Extended Data Fig. 3c).

## Discussion

Determination of accurate pharmacological parameters for a drug requires knowledge of the actual concentrations of the given drug that the receptor and/or tissue is exposed to. Particularly for class A G protein-coupled receptors (GPCRs), the lipophilic nature of many of the ligands means that local membrane interactions, and potentially re-binding effects, could have a significant influence on the local drug concentrations and therefore influence the observed pharmacology. In this study, for the first time, we directly measure concentrations of a fluorescent β_2_-adrenergic receptor ligand, BY-propranolol, in the immediate vicinity of a cell monolayer. Not only is the drug present in higher concentrations in the near vicinity of the cell membrane, but this is amplified further by the presence of the β_2_-adrenoceptor.

**Figure 4:**
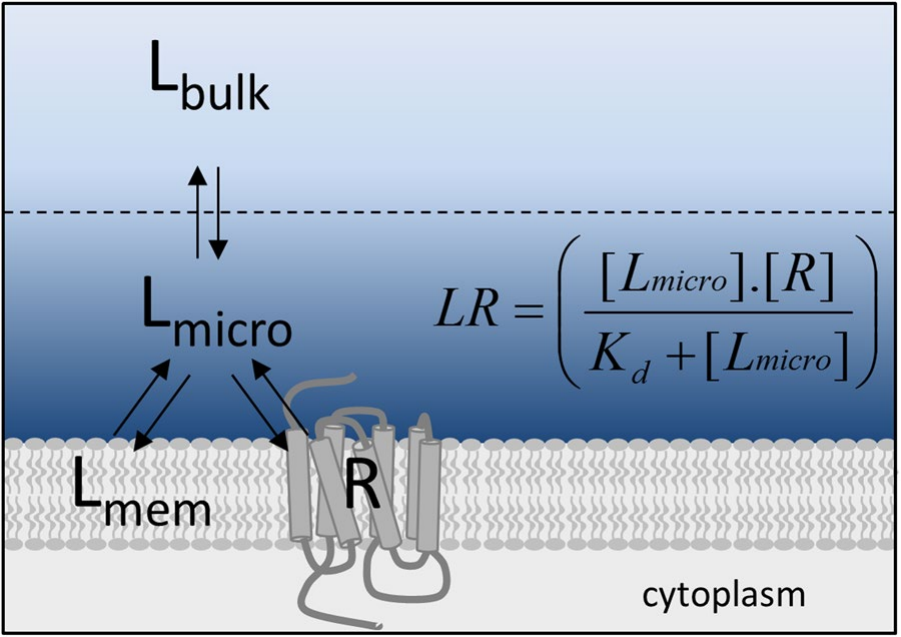
Schematic representation of increased local ligand concentrations in the immediate vicinity of the cell membrane and target receptors compared to the bulk aqueous phase. A ligand concentration gradient may potentially be caused by ligand interactions with both cell membrane components and target receptors (R). To account for heterogeneous ligand distribution in the determination of pharmacological parameters, higher local ligand concentrations (L_micro_) may be used, as shown here for affinity (K_d_) calculations.

In this study we used a BY-propranolol concentration reflecting its affinity (1.8 nM; 1 × K_D_) determined from equilibrium and kinetic binding calculations that assume a homogeneous distribution of ligand in the assay volume. However, our data have clearly shown that this assumption does not hold true and that the actual BY-propranolol concentrations the target receptors are exposed to are in fact much higher than the added concentration. Using the BY-propranolol concentration measured nearest the cell membrane in the microenvironment of the receptor (Fig. 4) yields a 25-fold lower (i.e. 45 nM) “true affinity” of BY-propranolol. Importantly, this concentration might possibly still be higher directly above the extracellular space of the receptors. We have recently estimated between 4 and 3,000-fold lower “true affinity” values for a range of β_2_-adrenoceptor ligands^5^, although these were based on their membrane partition coefficients (logK_IAM_), which reflect direct ligand interactions with membrane phospholipids.

It is interesting to consider what the ligand concentrations in the cell membrane might be, although these are technically challenging to determine using FCS due to differences in molecular brightness of BODIPY-labelled fluorescent ligands in aqueous compared to membrane environments^20^ and limitations of the FCS dynamic detection range for high ligand concentrations^11^. With these limitations in mind we have attempted to estimate ligand concentrations within the membrane, and our calculations suggest a time-dependent increase in BY-propranolol concentrations in the cell membrane of both receptor expressing and non-receptor expressing cells (Extended Data Fig. 4), with concentrations of 734 ± 137 nM (n = 9) and 835 ± 127 nM (n = 6) after 2 hours, respectively. These much higher membrane concentrations of ligand could potentially contribute further to the micro-pharmacokinetic profile of ligands that access their receptors via lateral diffusion in the membrane^2,21,22^, but are unlikely to be directly available to bind the β_2_-adrenoceptor as β-adrenoceptor ligands have been proposed to access their target receptor via its extracellular space in the aqueous solution^9^. It is therefore more likely that β-adrenoceptor ligands feed into the aqueous solution from a membrane sink, a mechanism that has previously been described in the ‘diffusion microkinetic model’^23^, and provides one possible explanation for the increasing concentrations observed over time at 2 µm above membranes of both receptor expressing and non-receptor expressing cells.

Interestingly however, BY-propranolol concentrations increased significantly over time only above membranes of receptor expressing cells, further supporting the role of the target receptor in concentrating ligand around its extracellular space. The influence of the receptor on local ligand concentration becomes an important notion, especially when considering different receptor expression levels not only in different tissues^24^ and cells^25^, but also in microdomains of a single cell^26,27^. It is widely appreciated that receptors are not uniformly distributed in cell membranes, and that they are associated with different effector and scaffolding proteins in different microdomains^26,28^, creating micro-environments of different potential ligand interactions. Our findings suggest that the presence and magnitude of a ligand concentration gradient may be influenced by the level and types of possible ligand interactions, which will depend on the physicochemical properties^5^ (e.g. charge, lipophilicity) of the ligand as well as the composition of the surface. Ligand interactions with membrane components such as phospholipids and sphingolipids have been described^4,7,8,21,29^, and provide additional interactions compared to those available in the bulk aqueous phase driving an increased local ligand concentration. Multicellular environments *in vitro* and *in vivo* will provide a much more complex level of ligand interactions resulting in unique micro-pharmacokinetic profiles for each ligand-receptor-cell micro-environment that may contribute to tissue-specific pharmacology for a given ligand-receptor complex.

As such, there is a clear need to develop approaches that allow better understanding of a ligand’s micro-pharmacokinetic profile and estimation of its local concentrations in a given micro-environment. Incorporation of these properties into current analyses of pharmacological characteristics has the potential to allow more accurate and therefore more physiologically relevant assessment of mechanisms of actions of ligands, and to drive drug design and optimisation strategies in early stages of drug discovery programs. For example, increased ligand-membrane interactions are thought to contribute to the long duration of action of β_2_-adrenoceptor agonists^23,30^ and may present a desirable feature in the design of new inhaled drugs. Furthermore, targeted drug design that takes into account ligand interactions in the multicellular environment of the target tissue may optimise tissue-specific pharmacology, maximising therapeutic effects and minimising unwanted side effects.

## Methods

### Cell culture

CHO-K1 cells (negative in PCR-based mycoplasma test) were stably transfected with cDNA encoding the human wild-type β_2_-adrenoceptor C-terminally tagged with the green fluorescent protein (GFP) to generate a mixed population CHO-β_2_GFP cell line (DNA sequencing verified the receptor sequence). Transfection was achieved using Lipofectamine (Life Technologies) according to the manufacturer’s instruction, and subsequent selective pressure (1 mg/mL G418) for 2–3 weeks. Following this, CHO-β_2_GFP cells were grown at 37 °C in CHO growth medium (phenol-red free Dulbecco’s modified Eagle’s medium/nutrient mixture F12 (Sigma-Aldrich) containing 10% (v/v) fetal bovine serum and 2 mM L-glutamine) in a humidified 5% CO_2_/95% air atmosphere.

### BODIPY630/650-PEG8-*S*-propranolol binding studies

BODIPY630/650-PEG8-*S*-propranolol (Compound 18a, ref.^10^) binding properties were determined in time-resolved Förster resonance energy transfer (TR-FRET) experiments using the Tag-lite® technology (Cisbio Bioassays). For saturation binding assays, membranes of HEK293 cells containing Lumi4®-Terbium labelled SNAP-tagged β_2_-adrenoceptors (HEK-ssβ_2_LT; 1 µg per well) were incubated in 384-well OptiPlates (PerkinElmer) at room temperature in binding assay buffer (Tag-lite® assay buffer (Cisbio Bioassays), 0.1% (v/v) pluronic acid, 100 µM GTPγS) with a range of BODIPY630/650-PEG8-*S*-propranolol concentrations (0.001–300 nM) in the absence and presence of 10 µM ICI 118,551 for 2 hours with gentle agitation, to determine total and non-specific binding, respectively. In kinetic binding experiments, observed association rates (k_onob_) of four increasing BODIPY630/650-PEG8-*S*-propranolol concentrations were obtained to allow accurate determination of association (k_on_) and dissociation rates (k_off_). BODIPY630/650-PEG8-*S*-propranolol concentrations were incubated in 384-well OptiPlates (room temperature) in binding assay buffer in the absence (total binding) and presence (non-specific binding) of 10 µM ICI 118,551. Kinetic reactions were started by the addition of HEK-ssβ_2_LT membranes (1 µg per well), and binding levels were measured every 30 seconds for 1 hour. The PHERAstar® FS plate reader (BMG Labtech) was used to measure fluorescence intensity emissions at 620 nm (HEK-ssβ_2_LT) and 665 nm (BODIPY630/650-PEG8-*S*-propranolol) following excitation (3 flashes per well) of Lumi4®-Terbium labelled SNAP-tagged β_2_-adrenoceptors at 337 nm using a Homogeneous Time-Resolved Fluorescence (HTRF) module. A TR-FRET ratio of 665 nm/620 nm fluorescence emissions (multiplied by 10,000) was calculated, and non-specific binding was subtracted from total binding to determine specific BODIPY630/650-PEG8-*S*-propranolol binding levels.

### Fluorescence correlation spectroscopy (FCS)

CHO-β_2_GFP cells were seeded into 8-well Labtek borosilicate chambered-cover glass plates (Nalgene Nunc International, Fisher Scientific) two days prior to experimentation, and grown to 50% confluency in CHO growth medium. On the day of the experiment, cells were equilibrated at 22 °C in HEPES buffered saline (HBS)^31^, and then exposed to 1.8 nM BODIPY630/650-PEG8-*S*-propranolol, before FCS measurements were performed on a Zeiss LSM510NLO Confocor 3 confocal microscope (Carl Zeiss, Jena) using a 40 × 1.2NA water immersion objective lens at 22 °C. The FCS measurement volume for the beampath used in these experiments was calibrated using 10 × 10 s data collection of a 10 nM Cy5-NHS ester solution on each experimental day^31^. For cell measurements, the confocal volume was positioned in x-y over a single CHO-β_2_GFP cell using a confocal image of GFP fluorescence (488 nm excitation, BP505/560 nm emission). Subsequent z-scanning was performed using ~0.03 kW/cm^2^ 633 nm helium-neon excitation to allow positioning of the confocal volume 2 µm above the peak fluorescent intensity of the upper cell membrane. FCS measurements of 30 seconds with ~2.4 kw/cm^2^ 633 nm excitation (LP650 nm emission) were taken 2–200 µm above the upper cell membrane, moving upwards in 1, 2, 10 and 50 µm increments as determined by the microscope harmonic drive within Zeiss AIM 3.5 software, following 15, 30, 60, 90 and 120 min BODIPY630/650-PEG8-*S*-propranolol incubation at 22 °C. In control areas of no cells FCS measurements were taken every 1 µm from 3–7 µm above coverslip (reaching equivalent average height of upper cell membranes), before following the same increments and distances measured in cell experiments. In antagonist experiments, cells were pre-incubated with 550 nM ICI 118,551 for 10 min (22 °C) prior to BODIPY630/650-PEG8-*S*-propranolol addition. Confocal imaging and FCS measurement settings were kept constant for all experiments.

### Data analysis and statistical procedures

Using Prism 6.0 (GraphPad Software) BODIPY630/650-PEG8-*S*-propranolol saturation binding data were analysed and association data were globally fitted as previously described^32^.

FCS analysis and data fitting was performed using Zen2010 software (Carl Zeiss, Jena). The measurement volume was calculated for each separate experiment using the measured dwell time (τ_D_) and structure parameter values from the Cy5-NHS ester solution calibration measurements, along with its literature diffusion coefficient (D = 3.16 × 10^−10^ m^2^/s) as previously described^31^. BODIPY630/650-PEG8-*S*-propranolol autocorrelation curves obtained from measurements in aqueous solution were fitted to a simple model describing one freely diffusing 3D diffusion component. For measurements within the cell membrane environment a model was used with one 3D diffusion component (free ligand) and one or two 2D diffusion components, as necessary (membrane bound ligand). For these fits, the dwell time of the 3D component was fixed to that of BODIPY630/650-PEG8-*S*-propanolol determined in aqueous solution experiments performed on the same day (τ_D_ ~ 140 µs). To correct particle number values in membrane reads for differences in the BODIPY630/650-PEG8-*S*-propranolol quantum yield in aqueous solution compared to the membrane, molecular brightness values were determined in these two environments by performing photon counting histogram analysis in an aqueous solution of BODIPY630/650-PEG8-*S*-propranolol (10 µm above membrane, component 1) and on the upper cell membrane (component 2) following 15 min incubation with 10 nM BODIPY630/650-PEG8-*S*-propranolol (1 × 60 s read, 1.0 kW/cm^2^ laser power), and a brightness correction ratio of 9.1 (component 2/component 1) was defined. BODIPY630/650-PEG8-*S*-propranolol concentrations and diffusion coefficients were determined using the calculated confocal volume from FCS calibration measurements for that day’s experiment. The confocal volume positioned at the cell membrane spans the membrane encompassing intra- and extracellular spaces in addition to the membrane. Ligand concentrations in the membrane were estimated from the particle number of the membrane component 2 in the autocorrelation fit of membrane measurements, and an estimated volume of a cylinder with an area of the FCS volume beam waist and the height of the membrane bilayer (5 nm). FCS measurements on one single cell per experimental condition were performed on one experimental day and as such represent one *n* number in this study.

Statistical analysis was performed on mean ± s.e.m. data using Graphpad Prism 6.0, with *P* < 0.05 representing statistical significance. T-test analysis was used to compare ligand concentrations to a fixed value (1.8 nM of BY-propranolol used in experiments), two-way ANOVA followed by Dunnett’s multiple comparison test was used to compare ligand concentrations from a range of distances above the cell membrane to the ligand concentration in the bulk aqueous phase (200 µm above membrane), and two-way ANOVA followed by Tukey’s multiple comparison test was used to compare ligand concentrations obtained under different experimental conditions for each distance.

### Data availability

All data supporting the findings of this study are available from the corresponding author on request.

## Electronic supplementary material

Supplementary information
