# Supplementary material for: Micro-pharmacokinetics: Quantifying local drug concentration at live cell membranes
Source: Sci Rep. 2018 Feb 22;8:3479. doi: 10.1038/s41598-018-21100-x (PMC5823863; doi:10.1038/s41598-018-21100-x)
Supplement: Supplementary file 1 — Supplementary information [file 41598_2018_21100_MOESM1_ESM.docx]

**Supplementary Information**

**Title**

Micro-pharmacokinetics: Quantifying local drug concentration at live cell membranes

**Authors**

Karolina Gherbi^1,3^, Stephen J Briddon^1,2^, Steven J Charlton^1,2,3^

**Affiliations**

^1^Division of Pharmacology, Physiology and Neuroscience, School of Life Sciences, Medical School, University of Nottingham, Queen’s Medical Centre, Nottingham, NG7 2UH, UK.

^2^Centre of Membrane Proteins and Receptors, Universities of Birmingham and Nottingham, The Midlands, UK.

^3^Excellerate Bioscience Ltd, MediCity, Nottingham, NG90 6BH, UK.

**Extended Data Fig. 1**
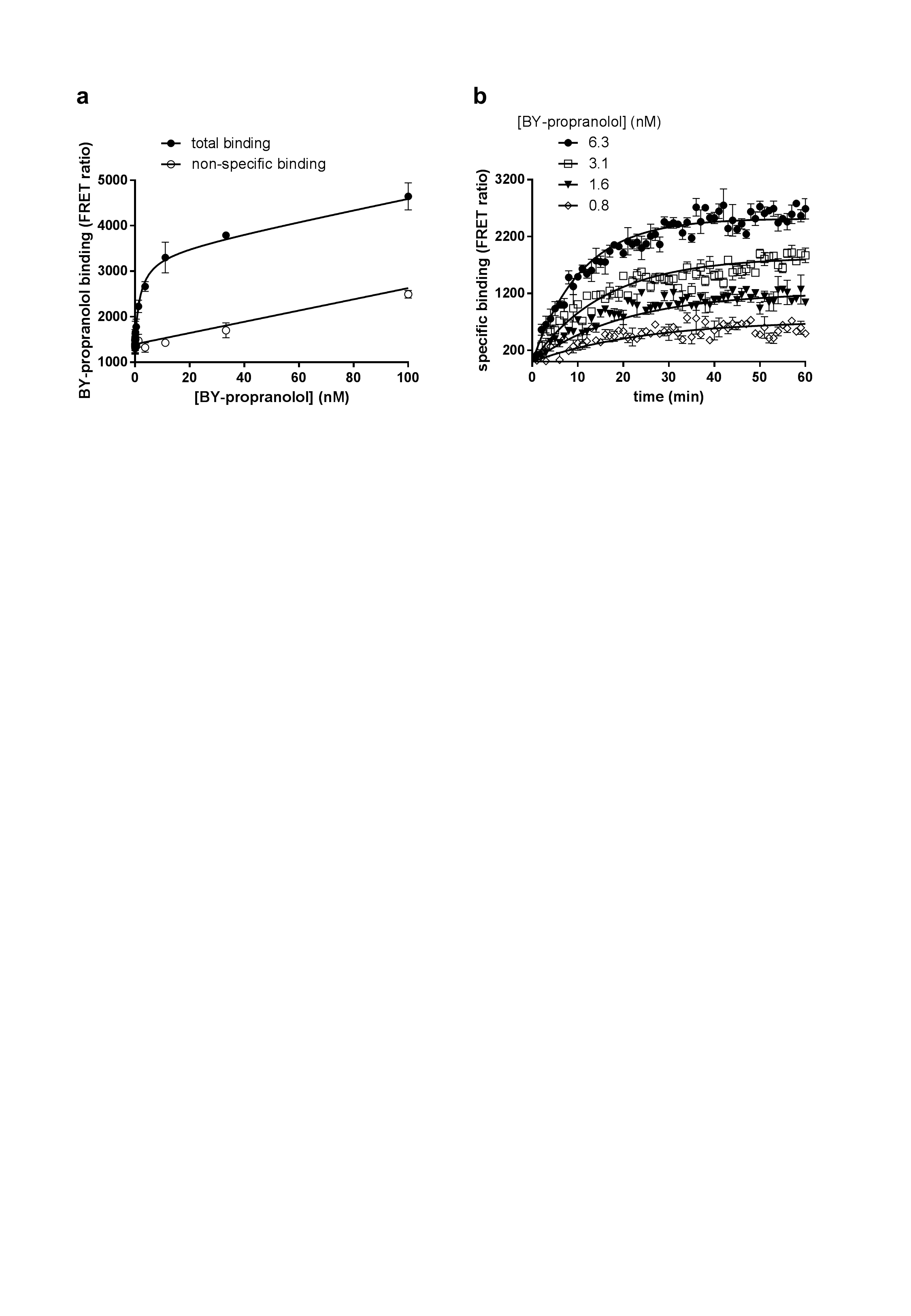


**FRET binding parameters of BY-propranolol determined in membranes of HEK cells expressing SNAP-tagged β_2_-adrenoceptors.** **a**, Total and non-specific BY-propranolol binding measurements determined a BY-propranolol binding affinity (pK_D_) of 8.66 ± 0.05 (n=3). Data are mean ± s.e.m. of a single experiment performed in triplicates, and are representative of three independent experiments. **b**, Specific binding association of 0.8, 1.6, 3.1 and 6.3 nM BY-propranolol yielded a BY-propranolol association rate constant (k_on_), dissociation rate (k_off_) and affinity (pK_D_) of 2.30 ± 0.29 x10^7^ min^-1^M^-1^ (n=4), 0.040 ± 0.006 min^-1^ (n=4) and 8.73 ± 0.04 (n=4), respectively. Data shown are mean ± range of a single experiment performed in duplicates, and are representative of four separate experiments.

**Extended Data Fig. 2**
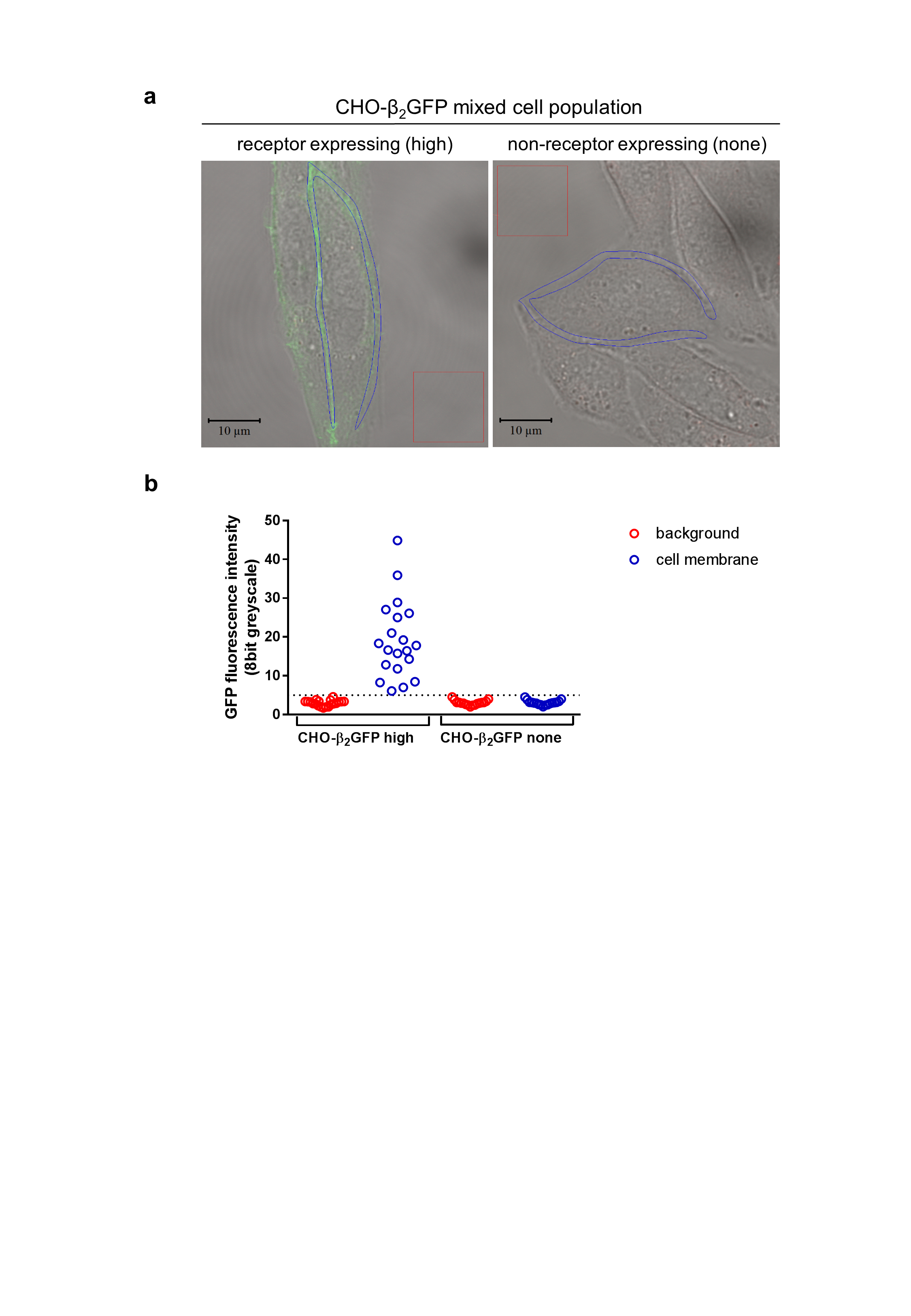


**Selection of receptor expressing and non-receptor expressing CHO-β_2_GFP cells based on GFP fluorescence.** **a**, Using the transmitted light image, regions of interests (ROIs) were drawn around the membrane (blue ROIs) of 37 CHO-β_2_GFP cells and background areas (green ROIs) in the same field of view. **b**, GFP fluorescence intensities measured in membranes of these cells and their respective background areas were compared, and based on detection of GFP fluorescence intensities above background (dotted line) 20 receptor-expressing (CHO-β_2_GFP high) and 17 non-receptor expressing (CHO-β_2_GFP none) CHO-β_2_GFP cells were identified in experiments in this study.

**Extended Data Fig. 3**


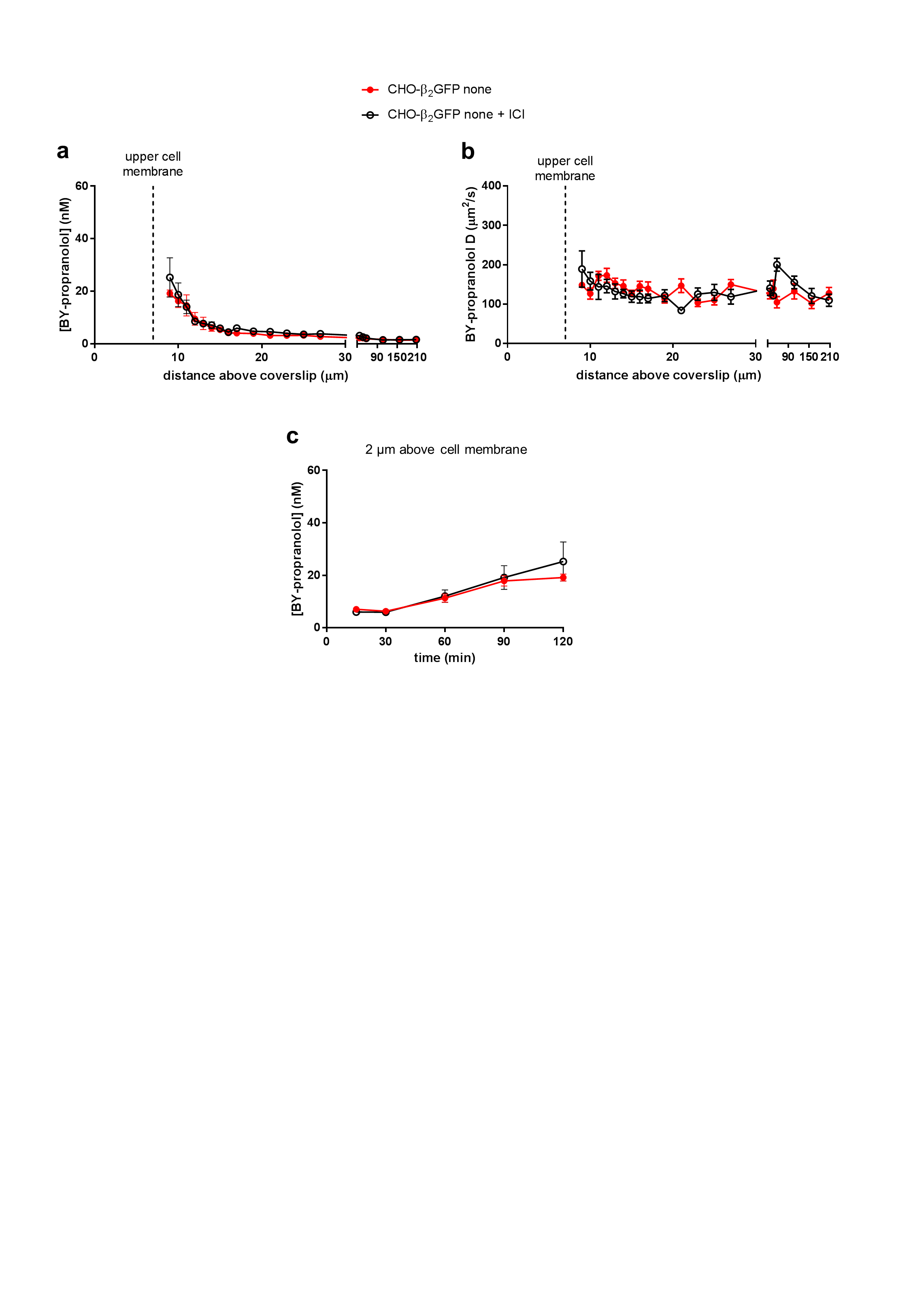
**Addition of ICI 118,551 to FCS experiments caused no detectable assay artefacts and non-specific effects.** **a**, **b**, Local concentrations (**a,c**) and diffusion coefficients (**b**) of BY-propranolol measured following 2 hours BY-propranolol incubation at a range of distances 2-200 µm (**a,b**) and following 15, 30, 60, 90 and 120 minutes BY-propranolol incubation at a fixed 2 µm distance (**c**) above membranes of CHO-β_2_GFP cells of no detectable receptor expression in the absence (n=10) and presence of 550 nM ICI 118,551 (ICI; n=7). Data shown are mean ± s.e.m. of *n* individual cells investigated on the same number of experimental days.

**Extended Data Fig. 4**
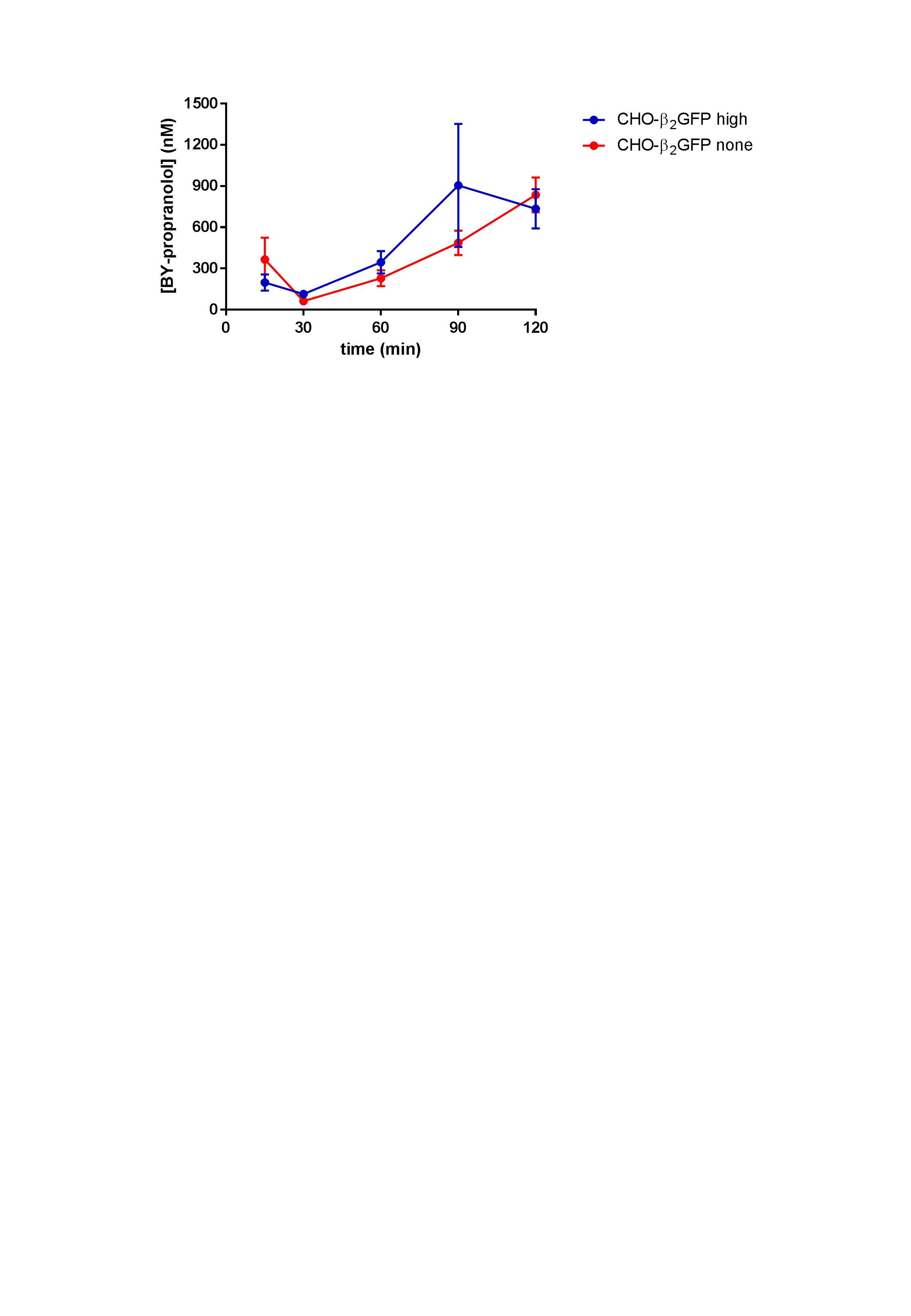


**Estimates of BY-propranolol concentrations in the membrane bilayer.** Concentrations of BY-propranolol were measured in membranes of receptor expressing and non-receptor expressing CHO-β_2_GFP cells over a time scale of 15 minutes to 2 hours, and data are mean ± s.e.m. of 9 and 6 individual cell membranes investigated on the same number of experimental days, respectively.
